# Isolation and identification of angiotensin I-converting enzyme inhibitory peptides derived from thermolysin-injected beef *M. longissimus*

**DOI:** 10.5713/ajas.18.0455

**Published:** 2018-08-24

**Authors:** Juhui Choe, Kuk-Hwan Seol, Hyun-Jin Kim, Jin-Taek Hwang, Mooha Lee, Cheorun Jo

**Affiliations:** 1 Department of Agricultural Biotechnology, Center for Food and Bioconvergence, and Research Institute of Agriculture and Life Science, Seoul National University, Seoul 08826, Korea; 2 National Institute of Animal Science, Rural Development Administration, Wanju 55365, Korea; 3 Department of Food Science and Technology, Gyeongsang National University, Jinju 52828, Korea; 4 Korea Food Research Institute, Wanju 55365, Korea; 5 Institute of Green Bio Science and Technology, Seoul National University, Pyeongchang 25354, Korea

**Keywords:** Beef, Injection, Thermolysin, Angiotensin I-converting Enzyme (ACE) Inhibitory Activity, Bioactive Peptides

## Abstract

**Objective:**

This study identified angiotensin I-converting enzyme (ACE) inhibitory peptides in beef *M. longissimus* injected with thermolysin (80 ppm) and stored for 3 days at 5°C.

**Methods:**

Crude peptides (molecular weight <3 kDa) were obtained from the thermolysin hydrolysate and separated into seven fractions. Fraction V showing the highest ACE inhibitory activity was further fractionated, yielding subfractions V-15, V-m1, and V-m2, and selected for superior ACE inhibitory activity. Finally, twelve peptides were identified from the three peak fractions and the ACE inhibitory activity (IC_50_) of each peptide was evaluated.

**Results:**

The Leu-Ser-Trp, Phe-Gly-Tyr, and Tyr-Arg-Gln peptides exhibited the strongest ACE inhibitory activity (IC_50_ values of 0.89, 2.69, and 3.09 mM, respectively) and had higher concentrations (6.63, 10.60, and 29.91 pg/g; p<0.05) relative to the other peptides tested.

**Conclusion:**

These results suggest that the thermolysin injection process is beneficial to the generation of bioactive peptides with strong ACE inhibitory activity.

## INTRODUCTION

The renin-angiotensin system (RAS) is one of the most important humoral vasoconstrictor and vasodilator mechanisms involved in blood pressure regulation. The system starts with the conversion of angiotensinogen to a prehypertensive hormone, angiotensin-I (Asp-Arg-Val-Tyr-Ile-His-Pro-Phe-His-Leu), by the action of renin secreted by the kidney [1]. Angiotensin-I is further converted to the active form of the hormone angiotensin-II (Asp-Arg-Val-Tyr-Ile-His-Pro-Phe), by the action of angiotensin-I converting enzyme (ACE). The Zn^4+^ metallopeptidase that removes the carboxy (C)-terminal dipeptide from decapeptide angiotensin I to generate the potent vasoconstrictor angiotensin II and inactivate the vasodilator bradykinin (Arg-Pro-Pro-Gly-Phe-Ser-Pro-Phe-Arg) or encephalitis [2]. The RAS can be regulated by ACE inhibitors [3].

Usually, ACE inhibitors are isolated naturally in mammals within the gastrointestinal tract during the normal metabolism of proteins [4]. However, a limited number of peptides can be released due to the specific cleavage sites of endogenous proteases that often display low productivity. For these reasons, the exogenous enzymatic hydrolysates from muscle protein have been used for the generation of ACE inhibitory peptides [5–7]. Porcine skeletal muscle proteins are hydrolyzed by eight proteases—thermolysin, proteinase K, proteinase E, ficin, papain, trypsin, α-chymotrypsin, and pepsin—and the ACE inhibitory activities of the hydrolysates have been measured [5]. Among the digests of the water-insoluble protein fraction prepared from muscle, a thermolysin digest demonstrated the highest activity among those that were tested. However, the majority of studies have been at the laboratory scale under optimum conditions, such as the use of high temperatures and ground meat.

The brine injection or marination process is commercially used for the quality enhancement of fresh meat [8]. Various ingredients including salt and synthetic or natural additives have been incorporated into brine, such as phosphate, fruit, or plant extracts containing proteases. A number of studies have reported that the injection process led to an instrumental or organoleptic enhancement of tenderness, juiciness, and the flavor of the meat [9,10]. A potent ACE inhibitory activity of sarcoplasmic and myofibrillar proteins extracted from beef injected with thermolysin and protease type XIII has been identified [11]. However, to our knowledge, there is little research on whole muscle injected with a proteolytic enzyme that could be utilized as a routine meat preparation practice in the home or in restaurants. Therefore, the aim of this study was to isolate and identify ACE inhibitory peptides from the hydrolysate of *M. longissimus* from beef generated by the injection of thermolysin followed by aging for 3 days at 5°C and to evaluate the ACE inhibitory activity of the identified peptides.

## MATERIALS AND METHODS

### Reagents

Thermolysin (T7902, Sigma, St. Louis, MO, USA), ACE (from rabbit lung), hippuryl-L-histidyl-L-leucine (HHL), hydrindantin, and lithium hydroxide were purchased from Sigma (USA). Acetonitrile (LC-MS grade) was purchased from Roth GmbH (Karlsruhe, Germany); L-Leucine from Aldrich Co. (Milwaukee, WI, USA); dimethyl sulfoxide (DMSO) from Merck (Darmstadt, Germany); ninhydrin from Riedel-de Haen (Seelze, Germany); and acetic acid and sodium hydroxide from Hayashi Pure Chemical Ltd. (Osaka, Japan).

### Meat sample preparation

**Figure 1:**
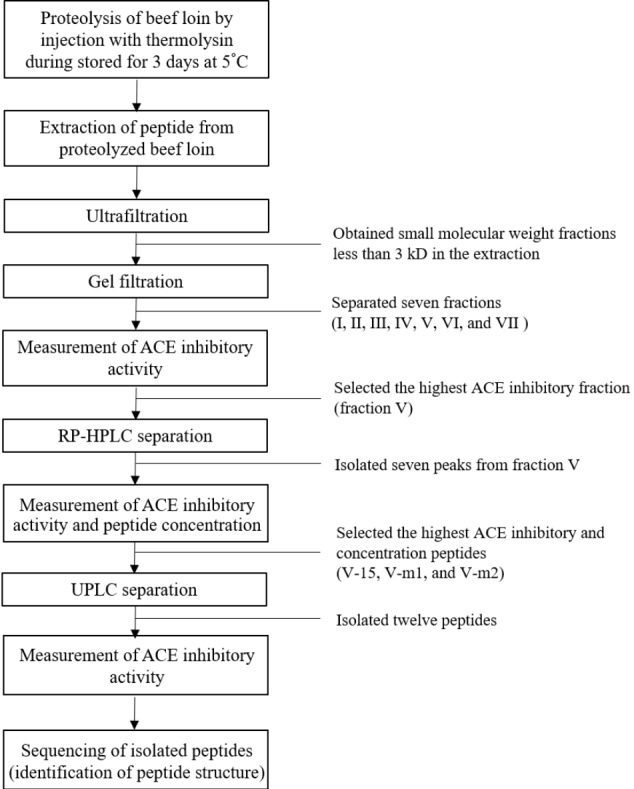
Flow chart for isolation and identification of angiotensin I-converting enzyme (ACE) inhibitory peptides.

At 24 h postmortem, loins (*M. longissimus*) were obtained from Hanwoo steer (quality grade 1, n = 3) slaughtered at a commercial slaughterhouse. The beef loins (200 g) were injected with 80 ppm of thermolysin and stored for 3 days at 5°C for enzymatic proteolysis of the muscle proteins. The use of thermolysin and its concentration were determined based on our preliminary study. The process for isolation and identification of ACE inhibitory was presented in Figure 1.

### Extraction and ultrafiltration

The peptides were extracted from the prepared beef samples as described in Jang and Lee [12]. Briefly, 5 g of each injected loin was homogenized in 20 mL of distilled water and boiled in a water bath at 95°C for 15 min to denature the enzymes. The homogenate was filtered and centrifuged at 10,000×*g* for 20 min at 4°C. The supernatant was collected and submitted to ultrafiltration at 4°C using a PM-10 membrane (10,000 molecular weight cut off [MWCO]; Amicon Co., Beverly, MA, USA) to remove high molecular weight peptides. The filtrates were then centrifuged at 4,000 *g* or 45 min at 4°C using an Ultracel 3K membrane (MWCO, 3,000; Amicon Co., USA). Prior to use, the membrane was activated with 10 mL of distilled water, and the remaining liquid was carefully removed. Separated crude peptide extracts were subsequently lyophilized and stored at −80°C until required for ACE inhibitory activity analysis and further separation.

### Gel filtration

The lyophilized crude peptides were dissolved in distilled water and again filtered through a Millipore membrane filter (0.45 μm) and applied to a column (Φ25×1,200 mm; Amersham Pharmacia Biotech, Little Chalfont, UK) saturated in P-2 resin. The eluent was deionized with distilled water with a flow rate of 0.8 mL/min. The fractions were collected at 15 min intervals with a fraction collector (Amersham Pharmacia Biotech, Uppsala, Sweden) and all fractions were subjected to absorbance measurements at 220 nm to confirm the peptide concentration. The fractions were collected on the basis of molecular weight and subsequently lyophilized prior to the ACE inhibitory activity measurements.

### Reversed-phase high-performance liquid chromatography

The highest ACE inhibitory fraction selected after gel filtration was dissolved in water containing 1% trifluoroacetic acid (TFA). For the quantitative analysis of ACE inhibitory peptides, reversed-phase high-performance liquid chromatography (RP-HPLC) was performed with a Jasco PU-980 gradient system with a Jasco photodiode array (PDA) detector at 220 nm (Jasco, Tokyo, Japan). The fraction solution (20 μL) was applied to a Vydac C18 reverse-phase column (4.5×25 cm; 5 Å, 300 μm pore size) and a solution containing 0.5% TFA, 0.5% acetonitrile (ACN), and 0.1% TFA was used for mobile phase at a flow rate of 1.0 mL/min. The seven major fractions were collected and subsequently lyophilized and the ACE inhibitory activity and concentration were measured. Among the seven fractions, the three fractions displaying strong ACE inhibition were applied to an ultra-performance liquid chromatography/quadrupole time-of-flight mass spectrometry (UPLC-QTOF-MS/MS) system to analyze the amino acid sequence of the peptides in the fractions.

### Small molecular peptide profiling by ultra performance liquid chromatography

An UPLC system (Waters, Milford, MA, USA) equipped with a binary solvent delivery system, an autosampler, and a PDA detector was used to analyze the peptides. An Acquity UPLC BEH C18 column (2.1×100 mm, 1.7 μm; Waters, USA) was equilibrated with water containing 0.1% TFA. HPLC fractions (5 μL) were injected into the column and eluted with an ACN gradient containing 0.1% TFA at a flow rate of 0.35 mL/min for 12 min. The absorbance of the eluent was detected at 214 nm by the PDA detector and QTOF-MS/MS.

### UPLC-QTOF-MS/MS peptide sequence analysis

The peptides from thermolysin hydrolysates of beef loin were separated and analyzed by UPLC-QTOF-MS/MS using a reverse phase C18 column (Waters, USA). The Q-TOF-MS/MS (Waters, USA) was operated in ESI positive mode. The capillary and sampling cone voltages were set at 2.78 kV and 26 V respectively. The desolvation flow was set to 700 L/h at a temperature of 300°C and the source temperature was set at 100°C. The QTOF MS data was collected in a 50 to 1,000 m/z range with a scan time of 0.2 s and an interscan delay time of 0.02 s. The MS/MS spectra of the peptides were obtained using a collision energy ramp from 10 to 45 eV. The accurate mass and composition of the precursor ions and fragment ions were calculated and sequenced using MassLynx software (Waters, USA) and the peptides were analyzed and sequenced by a BioLynx peptide sequencer (Waters, USA).

### Angiotensin I-converting enzyme inhibitory activity

ACE inhibitory activity determination was achieved using the spectrophotometric method described by Cushman and Cheung [13]. For each assay, 100 μL of HHL (12.5 mM in 0.05 M sodium borate buffer) was incubated at 37°C for 5 min. After incubation, 50 μL of bovine peptide extracts and 150 μL of ACE (peptidyldipeptide hydrolase from rabbit lung acetone extract) were added and the mixture incubated for 1 h. The enzymatic reaction was stopped by adding 250 μL of 0.5 N HCl. The hippuric acid generated by the action of the angiotensin-converting enzyme on HHL was extracted from the acidified solution into 1 mL ethyl acetate by vortex mixing for 15 s. This was centrifuged at 3,290×*g* for 10 min at 4°C, and a 0.7 mL aliquot of each ethyl acetate layer was transferred to clean tubes and evaporated by heating at 95°C for 20 min in a water bath. The hippuric acid was redissolved in 3 mL of 1 M NaCl, and the amount formed was determined by absorbance at 228 nm. The IC_50_ value, defined as the concentration of a peptide that inhibits 50% of the ACE activity, was determined by measuring the ACE inhibitory activity and the peptide contents of each of the extracts after regression analysis. ACE inhibitory activity (%) was determined using the equation:

ACE inhibitory activity (%)=[1-(S-SC)/(B-BC)]×100

where S and B are optical density (OD) values for the sample and blank, respectively, and SC and BC are the OD values for the sample control and blank, respectively.

### Chemical synthesis of peptides

The identified peptides were chemically synthesized by Peptron Ltd. (Daejeon, Korea) using the PenptrEX automatic peptide synthesizer and lyophilized for further confirmation of ACE inhibitory activity.

### Statistical analysis

The entire experiments were replicated three times individually and all the samples were analyzed in duplicate. All data were expressed as means±standard deviation. One-way analysis of variance was carried out to analyze the data using SAS program (SAS Institute, Cary, NC, USA). The treatment (fraction or peptide type) was the fixed effect in the model. Comparison of mean values was performed to find out the effect of treatment using Duncan’s multiple range tests. A value of p<0.05 was considered significant differences.

## RESULTS AND DISCUSSION

### Selection and isolation of angiotensin I-converting enzyme inhibitory peptides

**Figure 2:**
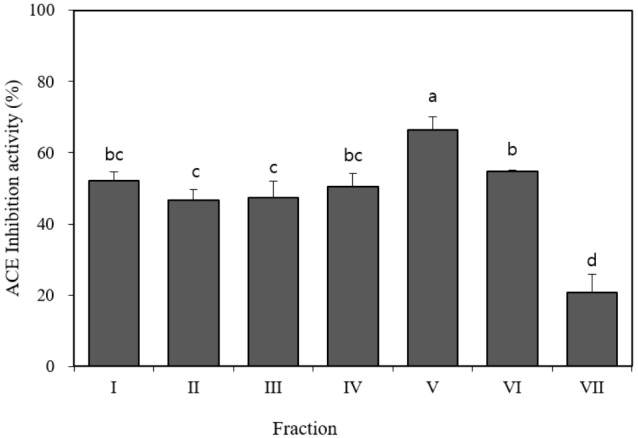
Angiotensin I-converting enzyme (ACE) inhibitory activity of seven different fractions separated from Hanwoo beef injected with thermolysin by P-2 gel filtration chromatography. Values are expressed as the means±standard deviation (n = 42). Error bars indicates SD. ^a–d^ Different letters represent ACE inhibition activity that was significantly different (p<0.05).

The thermolysin hydrolysates were filtered (MW <3 kDa) by ultrafiltration using PM-10 and Ultracel 3K membranes. After gel filtration, seven fractions were separated from the extract derived from beef loins injected with thermolysin and the ACE inhibitory activity of each fraction was evaluated at a concentration of 1 mg/mL. Fraction V inhibited ACE activity at the highest rate (66.5%), and the other five fractions (I, II, III, IV, and VI) achieved 50% inhibitory activity at a concentration of 1 mg/mL (Figure 2). This result corresponds with the results of other studies that have identified four gel-filtrated ACE inhibitory fractions with an optimal molecular weight having smaller than 3 kDa [12,14]. The fraction V with the highest ACE inhibitory activity was selected for further separation by RP-HPLC.

The major peak fractions (V-4, -5, -6, -7, -15, −m1, and −m2) were separated from fraction V by RP-HPLC. The most potent fractions (V-15, V-m1, and V-m2) that had the higher peptide concentrations and ACE inhibitory activities (Table 1) were selected and further analyzed by LC-MS/MS for sequencing.

### Isolated angiotensin I-converting enzyme inhibitory peptide sequencing

The total mass of the peptide mixture in each fraction was obtained from the mass detector, and the monoisotopic mass of each individual amino acid was subtracted to identify the exact sequence with an accurate mass. The MS/MS spectra of the potent peptides were displayed using a single positively charged ion (M+[H]^+^) and a total of twelve candidate peptides was identified from fraction V-15, V-m1, and V-m2 (Table 2). The highest ACE inhibitory activity was attributed to Leu-Ser-Trp (IC_50_ = 0.89 mM) from fraction V-15. The quantitative analysis of the three peptides with the highest ACE inhibitory activity that was generated by thermolysin-injected beef loin followed by 3 days of aging at 5°C—Leu-Ser-Trp, Phe-Gly-Tyr, and Tyr-Arg-Gln—was determined by LC-MS (6.63, 10.60, and 29.91 ng/kg respectively). These peptides were not detected in the non-injected control sample (data not shown). Many studies have found ACE inhibitory peptides derived from red meat, poultry, and fish muscle using proteolytic enzymes or processing, including aging or fermentation [6,15–17]. Also, previous study showed that dry fermentation of sausage during ripening produced ACE inhibitory peptides through the proteolytic activity of endogenous proteinases combined with that of microbial enzymes [18,19].

There are many reported protein structure-function studies that use different protein sources. Previous research has demonstrated that ACE inhibitory peptides have some common characteristics: i) The C-terminal tripeptide sequence strongly affects binding to ACE even though the underlying mechanism has yet to be completely established [20]; ii) C-terminal hydrophobic amino acids can effectively bind to the ACE active site [21]; iii) Di- or tripeptides with aromatic or branched-chain amino acids at the N-terminus and Tyr, Phe, Trp, or Pro at the C-terminus have interactions with ACE active site amino acid residues due to their strong affinity towards the ACE active site [7,22–24]. In the present study, Leu-Ser-Trp, the peptide with the highest ACE inhibitory activity (IC_50_ = 0.82 mM) had an aromatic C-terminal Trp, corresponding with the results of another study [25]. Additionally, aromatic or branched-chain aliphatic amino acids including Leu, Ile, Ala, Trp, and Met, contribute to potent ACE inhibition, playing an important role in ACE binding [7,25,26]. The peptide Phe-Gly-Tyr that had an IC_50_ of 2.69 mM for ACE inhibitory activity, consists of an aromatic amino acid at the N-terminus and Tyr at the C-terminus. Tyr-Arg-Gln also consists of an aromatic amino acid at the N-terminus. In other words, the three amino acid-sequenced peptides (Leu-Ser-Trp, Phe-Gly-Tyr, and Tyr-Arg-Gln) derived from thermolysin-injected beef with the greatest potential for use as ACE inhibitory peptides have highly similar sequence properties with peptides with well-established ACE inhibitory activity [27,28]. From the present study, Tyr-Gly exhibited lower ACE inhibitory activity in comparison with larger peptides (tri-, tetra-, penta-, or hexapeptides), except for Leu-Trp-Gly, despite their smaller size. This may be attributable to the absence of common ACE inhibitory peptide properties: C-terminal Tyr or hydrophobic amino acid or an N-terminal aromatic or branched-chain amino acid. This indicates that both the length of the amino acid chain and its composition is important for bioactivity and bioavailability as a functional inhibitory peptide [29].

Toopcham et al [7] reported that the tripeptide Met-Cys-Ser, containing the branched-chain amino acid Met at the N-terminus, exhibited higher ACE inhibitory activity than that of tetrapeptide Ala-Leu-Ser-Cys. Jang et al [12] reported that the ~3 kDa peptide had the highest ACE inhibitory activity of the peptides derived from beef rump hydrolysates. Furthermore, containing only a few amino acid residues, these peptides are able to cross the digestive epithelial barrier and reach blood vessels, allowing them to reach peripheral organs and have beneficial effects for the organism [29]. Because small peptides are more easily absorbed in the intestinal tract than larger peptides [30] and peptides containing proline are generally resistant to enzymatic digestion [31], it is expected that the peptides produced from thermolysin-injected beef loin will have sufficient antihypertensive activities *in vivo*.

## CONCLUSION

**Figure 3:**
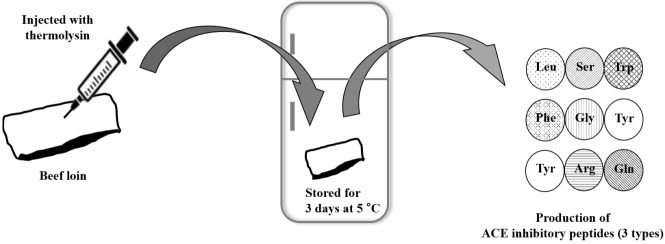
Schematic diagram of the production of angiotensin I-converting enzyme (ACE) inhibitory peptides from beef loin. Injection of thermolysin followed by 3 days refrigerated storage at 5°C increased ACE inhibitory activity of beef loin, which might be beneficial for human consumption.

In the present study, strong ACE inhibitory peptides—Leu-Ser-Trp, Phe-Gly-Tyr, and Tyr-Arg-Gln—were produced and identified from thermolysin-injected beef. Based on results through ultrafiltration, gel filtration, RP-HPLC, and UPLC-QTOF-MS/MS, injection of thermolysin into beef followed by 3 days refrigerated storage may increase ACE inhibition activity. Consequently, the consumption of the beef would be more beneficial for human health (Figure 3). Further research on ACE inhibition by the identified peptides *in vivo* will be a necessary step for their future use in the meat and meat products industry as functional biomaterials or a healthy dietetic method.

## Figures and Tables

**Table 1:** Peptide concentration and angiotensin I-converting enzyme (ACE) inhibitory activity of RP-HPLC fractions at 1 mg/mL after gel filtration (fraction V)

Fraction	Concentration (μg/mL)	ACE inhibitory activity (%)
V-4	ND	ND
V-5	ND	ND
V-6	ND	ND
V-7	ND	ND
V-15	903.5±5.4^b^	66.0±0.1^b^
V-m1	570.0±18.5^c^	46.4±0.3^c^
V-m2	1,664.1±29.8^a^	68.0±0.9^a^

RP-HPLC, reversed-phase high-performance liquid chromatography.

Values are expressed as the means±standard deviation (n = 3). ND, not detected.

a–c Different letters within different fractions were significantly different (p<0.05).

**Table 2:** The angiotensin I-converting enzyme (ACE) inhibitory activity (IC50) of chemically synthesized peptides

Source fraction	Peptide	IC_50_ (mM)
V-m1	Tyr-Gly	12.68
	Tyr-Tyr	8.60
V-m2	Tyr-Phe-Asn-Glu	7.62
V-15	Val-Ser-Val	9.08
	Leu-Trp-Gly	24.61
	Phe-Gly-Tyr	2.69
	Val-Ser-Ser-Val	8.49
	Leu-Ser-Trp	0.89
	Met-Ala-Asp-Ala	5.92
	Tyr-Arg-Gln	3.09
	Gln-Pro-Ser-Gly-Ser-Gln	8.78
	Leu-Trp-His- His-Thr	5.21

IC_50_ value was defined as the concentration of inhibitor required to inhibit 50% of the ACE activity.
